# Cancer Carepartners: Improving patients' symptom management by engaging informal caregivers

**DOI:** 10.1186/1472-684X-10-21

**Published:** 2011-11-25

**Authors:** Maria J Silveira, Charles W Given, Kemp B Cease, Alla Sikorskii, Barbara Given, Laurel L Northouse, John D Piette

**Affiliations:** 1 Center for Clinical Management Research, Veteran Affairs Medical Center, Ann Arbor, MI, USA; 2 Department of Internal Medicine, University of Michigan, Ann Arbor, MI, USA; 3 Center for Family Care, Michigan State University, East Lansing, MI, USA; 4 Division of Hematology & Oncology, Veteran Affairs Medical Center, Ann Arbor, MI, USA; 5 Department of Statistics and Probability, Michigan State University, East Lansing, MI, USA; 6 School of Nursing, University of Michigan, Ann Arbor, MI, USA

## Abstract

**Background:**

Previous studies have found that cancer patients undergoing chemotherapy can effectively manage their own symptoms when given tailored advice. This approach, however, may challenge patients with poor performance status and/or emotional distress. Our goal is to test an automated intervention that engages a friend or family member to support a patient through chemotherapy.

**Methods/Design:**

We describe the design and rationale of a randomized, controlled trial to assess the efficacy of 10 weeks of web-based caregiver alerts and tailored advice for helping a patient manage symptoms related to chemotherapy. The study aims to test the primary hypothesis that patients whose caregivers receive alerts and tailored advice will report less frequent and less severe symptoms at 10 and 14 weeks when compared to patients in the control arm; similarly, they will report better physical function, fewer outpatient visits and hospitalizations related to symptoms, and greater adherence to chemotherapy. 300 patients with solid tumors undergoing chemotherapy at two Veteran Administration oncology clinics reporting any symptom at a severity of ≥4 and a willing informal caregiver will be assigned to either 10 weeks of automated telephonic symptom assessment (ATSA) alone, or 10 weeks of ATSA plus web-based notification of symptom severity and problem solving advice to their chosen caregiver. Patients and caregivers will be surveyed at intake, 10 weeks and 14 weeks. Both groups will receive standard oncology, hospice, and palliative care.

**Discussion:**

Patients undergoing chemotherapy experience many symptoms that they may be able to manage with the support of an activated caregiver. This intervention uses readily available technology to improve patient caregiver communication about symptoms and caregiver knowledge of symptom management. If successful, it could substantially improve the quality of life of veterans and their families during the stresses of chemotherapy without substantially increasing the cost of care.

**Trial Registration:**

NCT00983892

## Background

Cancer is a prevalent problem that causes much suffering among patients and families. Most interventions to improve symptom control require patients to engage in activities such as managing medications, altering diets, or accessing outside resources that may be beyond their reach due to limitations in physical and mental functioning. Social network members inside and outside of the patient's household can assist with symptom monitoring, medication adherence, managing clinical encounters, and coping emotionally. Hogan et al found that among 92 studies meeting their entry criteria, 73 reported some benefit of social support provided by friends and families to patients with chronic conditions [1]. Unfortunately, cancer caregivers often lack the skills and resources they need to help the patient manage their treatment and the negative consequences of their disease and its management.

Few formal programs of supportive cancer care are designed to engage both patients and caregivers [2-11]. Among programs that have been evaluated, the majority of studies have had serious methodological flaws or limited evidence of effectiveness [8,12]. Northouse and colleagues examined the effects of three nurse home visits and two follow up nurse calls over four months to patients with prostate cancer and their spousal caregivers, with the goal of improving caregiver involvement, attitudes, coping, and quality of life [13]. Results showed some improvements in patient outcomes (better spousal communication and less uncertainty) and multiple improvement in caregiver outcomes (higher quality of life, self-efficacy, and communication; less symptom distress and negative appraisal of caregiving), with some effects sustained to 12 months follow-up. Given and colleagues tested the effects of a cognitive behavioral intervention administered by a nurse via telephone to assist patients undergoing chemotherapy to manage symptoms and assist caregivers to become involved in supporting symptom management. Patients receiving the intervention reported 34% and 30% lower symptom severity at 10 and 20 weeks, respectively [11]. Caregivers did not report any change in their involvement or distress, and some caregivers reported increased depressive symptoms [14,15].

The current protocol describes an ongoing randomized trial designed to address these gaps in the literature by developing and evaluating an intervention that provides cancer patients and caregivers with the information they need to make effective management decisions, decrease symptom burden, and improve patient and caregiver outcomes. The study was funded by the VA Health Services Research and Development Service and began October, 2009. The trial will be completed in August 2013.

The intervention uses a Web-enabled program to alert caregivers of patients' symptoms and provide them with a framework for identifying problems, receiving structured advice, and following up to assist the patient in manage symptoms while receiving chemotherapy. Specifically, the intervention includes weekly, automated telephonic symptom assessment with self-management support calls to patients paired with Web-based caregiver alerts that include customized advice. The study will enroll 300 patients with solid tumors undergoing chemotherapy identified from two Department of Veterans Affairs (VA) outpatient oncology clinics. Patients are recruited along with an informal caregiver willing to play a limited role in assisting the patient with symptom management. The impact of the intervention will be evaluated relative to a control arm in which patients receive automated telephonic symptom assessments and self-management advice without any involvement of their identified caregiver. Thus this is one of the few rigorously designed interventions that tests the value added impact of engaging informal caregivers in symptom management for patients with cancer. Specific study hypotheses are as follows:

*Hypothesis 1a: Patients receiving automated telephonic symptom assessment and management advice, with Web-based feedback to inform and engage a caregiver, will report less frequent and less severe symptoms at 10 and 14 weeks when compared to patients receiving automated telephonic symptom assessment and management advice alone. Intervention-group patients will also report better physical function, fewer outpatient visits and hospitalizations related to symptoms, and greater adherence to chemotherapy*.

*Hypothesis 1b. The effect of the intervention on symptom severity, physical functioning, and utilization of services will be partially mediated by improved patient self-efficacy and actualized social support*.

*Hypothesis 2a: Caregivers receiving the intervention will provide significantly more social support to patients at 10 and 14 weeks as evidenced by increases in patient-reported measures of social support when compared with caregivers who do not receive the intervention*.

*Hypothesis 2b: Caregivers receiving the intervention will report lower perceived caregiving burden and distress compared to caregivers in the comparison group*.

### Theoretical Model

**Figure 1:**
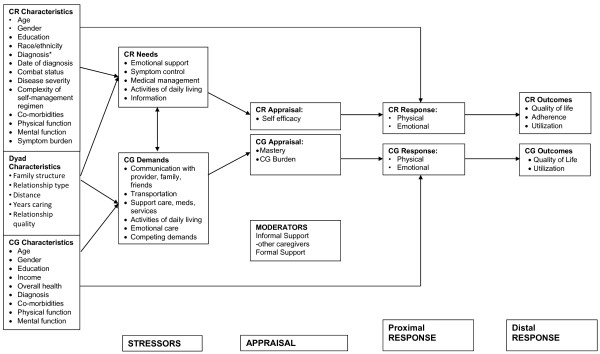
**Depiction of theoretical model**. CG = caregiver; CR = care recipient.

The study is based on the stress-coping model which identifies the processes influencing quality of life in patients with cancer (Figure 1) [16,17]. According to this model, when a cancer patient develops a need-e.g. a symptom-s/he is able to address the need to the extent that s/he is self-efficacious and functional. The system we are evaluating improves patient self-efficacy by providing patients with tailored information about what they can do to feel better. By activating an informal caregiver to support the patient, there is an additional source of information and encouragement to promote the patient's self-efficacy. How the patient uses the information provided during automated telephonic interactions determines his/her quality of life, ability to comply with chemotherapy, and utilization of services. The proposed intervention serves not only to improve patient access to information, but also to increase the speed of information transfer between patient and caregiver.

### Intervention Components

#### Automated Telephonic Symptom Assessment (ATSA)

ATSA is an automated telephonic system that calls patients at home to collect their symptom ratings via responses to recorded queries using their telephone's touchtone keypad. ATSA's questions consist of items from the MD Anderson Symptom Inventory modified for the telephone and validated in prior cognitive-behavioral studies of symptom management [15,18-21]. The ATSA system was developed based on prior research showing that patients with cancer and other serious chronic diseases can and will report reliable information during automated assessment calls, and that such calls may improve their self-care and health outcomes [22-25]. The duration and intensity of the intervention is premised on past work that suggesting that ATSA's benefits peak at 10 weeks [26].

The ATSA system calls patients weekly and asks them to rate the frequency and severity of eight core symptoms: pain, distress, fatigue, insomnia, nausea, poor appetite, dry mouth, and constipation. The choice of core symptoms is based on prior work by Given and colleagues showing that among patients undergoing chemotherapy, 85% experience at least one of these symptoms [11]. Patients are asked how many days during the last week they have experienced each symptom. When patients report a frequency of once per week or greater, the ATSA prompts patients to rate the severity of that symptom on a scale of 0 to10.

During the automated telephonic interaction, the ATSA system gives targeted advice for self-management depending upon the severity of the symptoms reported by the patient. For a severity of three or less, ATSA gives no advice. For a severity greater than three, ATSA refers the patient to the appropriate section of a printed "Symptom Management Toolkit" which the patient receives upon study enrollment. Details about the Toolkit are provided below. When a symptom is reported with a severity of seven or higher, ATSA not only refers the patient to the appropriate section of the Toolkit, but also recommends that the patient contact his/her VA oncology provider. The cut points of 3 and 7 were chosen based on severity scores shown to have similar levels of interference with functioning among patients with cancer [21]. If an ATSA call is not completed at the designated time, the system automatically attempts a repeat call twice at 15 minute intervals. If not completed that day, another call is attempted on the next day at the same time. ATSA stores data on patients' symptom scores as well as the number of attempted call assessments, date/time stamps for all call attempts, and the number of missed call attempts for the purposes of evaluating service usability.

#### The Symptom Management Toolkit

The Toolkit is a printed, patient guide for managing symptoms commonly seen in cancer, organized by symptom in a question/answer format. The Toolkit was developed by Charles W Given and Barbara Given for use in ATSA. Strategies included in the Toolkit are based on recommendations from the National Comprehensive Cancer Network, National Institutes of Health, Oncology Nursing Society, and other groups of nationally recognized experts, supplemented by literature gained from extensive reviews of MEDLINE, CINAHL, PsychInfo, and PubMed for evidence-based interventions [27-29]. The Toolkit has been successfully used by both patients and in-home caregivers to manage symptoms in patients undergoing chemotherapy in five prior studies [11,14,15,20,30].

#### Cancer CarePartners

**Figure 2:**
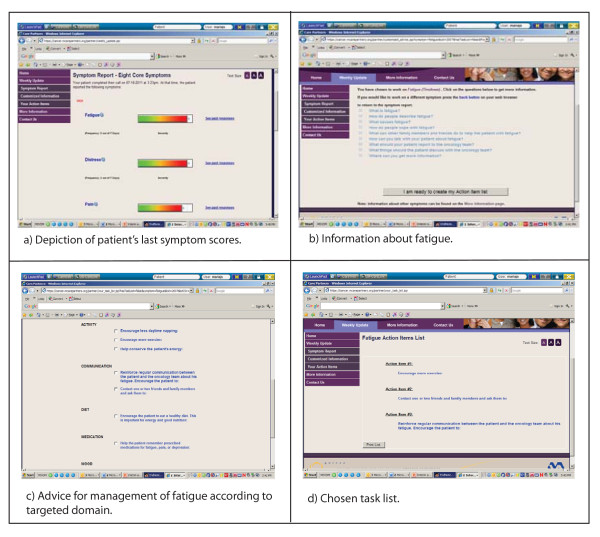
**Screen shots of Cancer CarePartners Web site**.

Cancer CarePartners is a Web based resource that draws information from the ATSA calls and uses that as well as a detailed set of caregiver support tools to provide caregivers with assistance in decision-making and instrumental assistance to improve patients' symptom management. Following each of patients' ATSA assessments, Cancer CarePartners alerts the caregiver about the patient's symptoms and helps the caregiver develop strategies for addressing the patient's needs. Cancer CarePartners was designed based on the problem-solving conceptual framework established by D'Zurilla and Nezu for family caregivers of people with physical illnesses [31]. In this framework, problem solving is seen as the rational and systematic construction of a solution through the use of four specific problem solving skills: 1) problem definition and formulation, 2) generation of alternatives, 3) decision making, and 4) solution implementation and verification. Table 1 summarizes how Cancer CarePartners develops these four skills in cancer caregivers; screenshots are shown in Figure 2.

**Table 1:** Cancer CarePartners Web site components and caregiver skills targeted.

**Targeted caregiver skills**		**Web site components**
		
Problem definition & formulation	**→**	Upon logging into the Cancer CarePartners web site, the caregiver sees a depiction of the patient's last symptom scores using horizontal thermometers (Figure 1a). Symptoms are presented in order of severity, with the most severe symptoms displayed at the top of the screen. Caregivers can select a symptom to access additional information about the symptom's cause and management (Figure 1b). Caregivers also can access reports showing trends in each symptom over time.
		
Generation of alternatives	**→**	The Web site asks caregivers to identify one symptom that they would like to help the patient work on that week. Upon choosing the symptom, the caregiver sees a list of recommendations suited to the patient's last assessment (Figure 1c). Advice is grouped by its targeted domain: namely, activity (i.e. exercise), communication, diet, medication, and mood. For example, if the patient reported dry mouth and the caregiver chooses to address that symptom with additional self-care assistance, they may receive advice such as "make sure the patient has a water bottle at his/her side at all times."
		
Decision making	**→**	Caregivers are asked to commit to trying three to five tasks for the week from the list of recommendations. To help caregivers make informed decisions about which recommendations to adopt, the Web site describes each task's purpose and what is involved, and provides examples for how to accomplish it.
		
Solution implementation & verification	**→**	After committing to 3-5 tasks, caregivers can print a list of their choices, or "Task List, " for future reference (Figure 1d). The next time the caregiver logs into the website, she is asked which of the tasks she attempted and whether the recommended action was helpful (Figure 1e). In addition to allowing caregivers to self-monitor the outcomes associated with their assistance, the data allow the investigative team to determine uptake of specific advice offered by Cancer CarePartners and caregivers' perceptions about its benefit.

## Methods

The intervention's efficacy is being evaluated using a randomized, controlled trial among patients undergoing a course of chemotherapy at two Department of Veterans Affairs (VA) oncology clinics located in Ann Arbor, MI and Fargo, ND. Three hundred patients with solid tumors undergoing chemotherapy and reporting at least one core symptom at a moderate level are being assigned to either 10 weeks of weekly ATSA contacts or 10 weeks of weekly ATSA contacts plus caregiver alerts and access to the Cancer CarePartners website. The duration and intensity of the intervention is premised on past work suggesting that ATSA's benefits peak at 10 weeks [11].

The goal of the study is to determine if, when compared to a comparison group receiving ATSA alone, patients who receive ATSA and whose caregivers receive access to Cancer CarePartners will report: significantly less total symptom severity; and secondarily improved physical functioning, adherence to chemotherapy, and more appropriate utilization of health services at 10 weeks. The study also will determine whether caregivers provide more social support and report less caregiver burden and distress.

Patients and caregivers will be surveyed at intake, 10, and 14 weeks. Further, we will track patient participation with ATSA and (in the experimental arm) caregiver use of the Website and social support provided, allowing for indicators of intervention dose to be incorporated into our analyses. Finally, all patients will receive routine oncology, hospice, and/or palliative care.

### Eligibility

Study patients must be 21 years or older, cognitively intact, English speaking, able to hear, and able to use the phone. They must have home access to a touch-tone telephone. They must have a solid tumor; for example, prostate, lung, colorectal, breast, non-Hodgkin lymphoma, or head/neck cancer. Patients may have new or recurrent disease in early or late stage. All patients must be within 10 weeks of initiating chemotherapy and, if they are being treated for recurrent disease, must have experienced a treatment free interval of 10 weeks between the new and past treatment. Patients may live alone or with others, with or without an in-home caregiver. Patients are excluded if they have been diagnosed with a hematologic malignancy or their treatment regimen involves bone marrow transplantation because such patients' symptom profiles differ from those of other cancer patients. Also, patients will be excluded if they (a) have untreated mental illness or cognitive impairment limiting their ability to participate with ATSA, (b) cannot identify at least one potential consenting caregiver, (c) are institutionalized or enrolled in hospice prior to enrollment, or (d) plan to discontinue receiving the majority of care from one of the study sites during the follow-up period.

For the purposes of this study, a potential caregiver *is defined as a person living inside or outside of the patient's household who has at least monthly contact with the patient, either by phone or in person, and is willing to help the patient manage his/her symptoms*. S/he must be 21 years or older, cognitively intact, English speaking, and able to hear and speak English for telephone interviews. S/he must have access to a telephone and computer with a high-speed Internet connection.

Caregivers need not be providing direct care prior to the study; indeed, a major motivation for this intervention is that many well-intentioned friends and family members would be willing to provide more cancer support, but currently do not do so because there is no structure in place to make that possible. Caregivers will be excluded if they, themselves, are on active treatment for cancer. They will also be excluded if they have poorly controlled mental illness, moderate cognitive impairment, or are unable to be interviewed by telephone.

### Recruitment & Randomization

Study staff review the schedules of participating chemotherapy infusion clinics weekly to identify potentially eligible patients. The list of potentially eligible patients is provided to staff oncologists for their approval prior to attempting recruitment.

Eligible patients are approached by research assistants after their second week of chemotherapy. Research staff explain the study to them, screen for eligibility, offer enrollment, and obtain informed consent. Patients agreeing to participate are asked to identify at least four potential caregivers using an algorithm based on the Norbeck Social Support Scale to determine the ranking of choices according to their involvement in the patient's life [32,33]. Research assistants then obtain contact information for the potential caregivers. Potential caregivers are recruited by research assistants by phone or in person if they accompany the patient to clinic. The research assistants explain the study, screen for eligibility, and obtain written informed consent. When a potential caregiver declines participation in the study, the research assistants contact the remaining potential caregivers on the list following the ranking provided by the patient. If no potential caregiver consents to participate, the patient is excluded from the study.

Patient participants are asked to identify preferred calling times for their ATSA calls. In addition, they are instructed on the use ATSA and rehearse an ATSA symptom assessment with the research assistant while in clinic. All patient participants are told to contact the project manager if they have any questions during their participation in the study and are given the telephone number for their respective oncology clinic for clinical issues not related to the study or when advised to do so by ATSA.

Basic sociodemographic and clinical data are kept on patients and caregivers who are ineligible for or refuse enrollment in order to understand the reach of the intervention in the broader population of eligible patients. In particular, we will describe the extent to which the requirement of caregiver Internet access serves as a barrier to recruitment among economically disadvantaged or rural patients.

After completing the baseline assessment, participating patients are randomized to either the control or intervention arm by a computer minimization program that balances the arms for type of cancer (lung vs. other) and the type of caregiver (in-home vs. out of home). The minimization procedure, known as 'adaptive randomization' balances trial arms according to the history of previous allocations. Arms are being balanced for lung cancer since patients with lung cancer have a symptom pattern distinct from patients with other solid tumors [34,35]. Arms are being balanced for type of caregiver because in-home caregivers have more access to the patient than do caregivers living at a distance and, hence, more opportunities for providing support.

### Comparison group

Patients randomized to the control arm of the study receive the Symptom Management Toolkit followed by weekly ATSA calls for 10 weeks. Caregivers in the control arm receive an email with a PDF copy of "What you need to know about cancer" from the National Cancer Institute (available from http://www.cancer.gov/cancertopics/wyntk/colon-and-rectal/page1). This booklet on cancer discusses possible causes, symptoms, diagnosis, treatment, emotional issues, and questions to ask the doctor.

### Intervention group

Patients randomized to the intervention arm receive the Symptom Management Toolkit and weekly ATSA calls for 10 weeks, as received by control-group participants. In addition, however, their caregiver receives weekly emails prompting them to log into the Cancer CarePartners website when their patient reports any core symptom severity of four or higher. The website informs caregivers of their patients' symptom assessment scores and provides them with advice for how to help, as described above. For the first 4 weeks in the study, staff monitor each caregiver's use of the website. When caregivers do not log into Cancer CarePartners within 48 hours of an assessment, staff telephone them to troubleshoot as needed.

### Data Sources

Data are being gathered from patient and caregiver surveys, ATSA, the Cancer CarePartners website, and medical record review.

#### Patient & caregiver surveys

Research assistants interview patients and caregivers by phone at baseline, 10 weeks post baseline, and 14 weeks post baseline. Patient surveys assess the study primary outcome (i.e. symptom severity) and secondary outcomes (i.e. physical and mental functioning, adherence to chemotherapy, and utilization of health services). Surveys also gather data on potential mediators of intervention effects (i.e. self-efficacy and actualized social support). Goals of the caregiver surveys are to measure caregiver outcomes (i.e. distress and burden). Patients and caregivers receive $15 for each completed survey.

#### ATSA

ATSA tracks call attempts, call outcomes (completed vs. incomplete), and reported symptom scores. The number of completed calls relative to call attempts will be used to describe patient uptake of the intervention as well as the dose received.

#### Cancer CarePartners website

The website tracks the number of email alerts sent to caregivers, numbers of log-in attempts, the web pages visited, the time spent per page, the tasks chosen, and caregivers' responses to the weekly task list survey. These data will be used to describe the usability of the website, as well as caregivers' choices of caregiving tasks from suggested options.

#### Chart review

A registered nurse reviews the patient's electronic medical record at week 14 or following withdrawal or death to assess covariates (e.g. comorbidities, supportive care received) and secondary outcomes (utilization, adherence with chemotherapy, and adverse events). The nurse reviews all inpatient and outpatient oncology notes written from four weeks prior to 14 weeks after informed consent. All modalities of cancer treatment and adjunct supportive care received during the observation period are noted (including consultations from palliative care services or referrals to hospice or home health nursing). Surgical procedures, the dose and duration of radiation treatment, and the dose and duration of chemotherapy (both recommended and actualized) will be noted, as will any medication changes (and reasons thereof).

### Description of Patient Measures

#### Summed symptom severity score

(source: patient baseline, 10 week, and 14 week surveys). A summed symptom score will be generated based on the sum of symptom severity scores (scored 0-10) across the 8 core symptoms.

#### Adherence to chemotherapy

(source: medical record audit). Adherence to chemotherapy will be determined by the total number of dose reductions, infusion delays, and agent discontinuations.

#### Physical functioning

(source: patient baseline, 10 week, and 14 week surveys) Physical functioning will be measured using the SF-36 summary measure for physical function which has an alpha reliability exceeding 0.80 [36].

#### Utilization of services

(source: medical record audit). The utilization of inpatient and outpatient services will be measured in terms of the number of cancer-related: hospitalizations, visits to emergency or urgent care, scheduled and unscheduled contacts with oncology (in person or by phone), and referrals to home health or hospice.

#### Self-efficacy

(source: patient baseline and 10 week surveys). The Cancer Behavior Inventory

(CBI) Version 2 will be employed to measure patient self-efficacy. The CBI has an internal validity of .94 and covers 7 aspects of self-management, including: maintenance of activity and independence, seeking and understanding medical information, stress management, coping with side effects of treatment, accepting cancer/maintaining positive attitude, affective regulation, and seeking support [37].

#### Social support

(source: patient baseline and 10 week surveys). Two dimensions of social support will be examined: actualized and perceived. Actualized social support will be measured using the Inventory of Socially Supportive Behaviors (ISSB) [38]. The ISSB is a 40 item measure that assesses the receipt of directive guidance (e.g. caregiver suggested the patient take some action), nondirective support (e.g. caregiver expressed concern), positive social exchange (e.g. the caregiver talked with patient about non-cancer related interests), and tangible assistance (e.g. caregiver drove patient to appointment). The ISSB has excellent internal consistency and good test-retest reliability (> 0.90) [38]. Perceived social support will be measured using the Social Provisions Scale (SPS). The internal consistency scores of the six subscales and total scale of the SPS are above .70 and .90 respectively.

### Description of Caregiver Measures

#### Caregiver burden

(source: caregiver baseline, 10 week, and 14 week surveys). The Caregiver Reaction Assessment (CRA) measures 5 dimensions of caregiver burden, including: caregiver esteem, lack of family support, impact on finances, impact on schedule, and impact on health. The CRA has good internal consistency (alpha 0.80-0.90) and a high degree of reliability [39].

#### Caregiver distress (source: caregiver baseline, 10 week, and 14 week surveys)

We will employ the Center for Epidemiological Studies-Depression Scale (CES-D) to measure depression in CarePartners. The CES-D is a scale widely used to measure depression in caregivers because of its ease of use (it has 20 items) and high reliability (alpha = 0.90) [40].

#### Mastery of caregiving

(source: caregiver baseline and 10 week surveys). Caregiver mastery will be measured using the problem solving skill subscale of the caregiver self-efficacy instrument by Zeiss; this subscale has a reliability of .83 [41].

### Quality control

Research assistants rehearse an ATSA call with every patient participant at the time of enrollment. They review call data from ATSA regularly to identify patients who do not respond to scheduled calls. When patients do not respond to multiple ATSA attempts over 48 hours (at any period throughout the 10 weeks of the trial), research assistants contact them to determine the reason; reasons for non-compliance are tracked. Support is offered to patients whose reason for noncompliance relates to technical issues.

Similarly, for the first 4 weeks on the study, caregivers' Web logs are reviewed to identify caregivers who either have not been able to sign-in or create a task list. Research assistants contact caregivers whose Web logs indicate noncompliance and call caregivers to identify the cause. Depending on the reason for the difficulty, research assistants offer to guide the caregiver by telephone during their next online session. Research assistants track all reasons for noncompliance with the website.

Patients and caregivers are not permitted to use ATSA or the website until the baseline survey is completed. Data is entered into a clinical data tracking system that alerts staff of survey due dates and incomplete data. The date the baseline survey is completed determines the dates for the 10 and 14 week surveys. Up to 10 attempts are made to contact each subject or caregiver for scheduling an interview. Abbreviated versions of all interviews are available for patients who have difficulty completing the entire survey. Patients and caregivers receive a $15 gift card for each survey they complete. Monthly updates with comments on completeness of data are maintained and reviewed by the Project Manager.

The success of randomization is monitored quarterly by comparing the demographic and clinical characteristics of patients across study arms. Clinical staff is blinded to the assignment arm, as are the analysts examining the data.

### Monitoring and Reporting of Adverse Events

The study includes several mechanisms for monitoring and identifying adverse events. First, symptom data from ATSA are reviewed weekly by the Principal Investigator (PI) who is a physician. When a patient reports multiple symptoms at severe levels (7 or above), the PI reviews the medical record to insure that the symptoms have been recognized by oncology staff. In the event that there are no progress notes indicating that the patient has been recently seen or contacted, the PI calls the oncology nurse case manager to alert them to the patient's reports.

When patients fail to respond to ATSA calls on two consecutive days, research staff calls the patient to ensure their safety. When patients cannot be reached, study staff informs the caregiver and oncology nurse case manager. Patients who are hospitalized are given the option to suspend their ATSA calls. Patients enrolling in hospice are given the option to come off study.

Subjects and clinicians have access to a toll-free number they can call with study-related problems. Caregivers have access to the research team's contact information through the website. Adverse clinical events are reported immediately to the clinician of record as well as the IRB. All deaths are immediately investigated by a physician not affiliated with the study in order to determine the cause.

### Data Analysis

#### Sample Size Calculation

Testing the primary outcome using two sided t-testing will require at least 224 dyads (patients and caregivers) to complete the study (112 per arm). This sample size affords 80% power to detect a difference as small as 0.33 standard deviations in mean summed symptom severity (Hypothesis 1) and social support (Hypothesis 2) between arms using 0.05 as the level of significance. This consistent with standards for clinical relevance and those seen in similar interventions [26,42]. Moreover, this sample size is sufficient to accommodate longitudinal analyses with repeated measures. To account for 25% attrition (consistent with prior trials of ATSA) and ensure that 112 dyads in each group are available for analysis, the goal is to enroll 300 dyads in the trial [26].

#### Preliminary Analysis

Graphical analyses will include: (1) examining distributions of the various numerical outcome variables using box plots and histograms to investigate skewness, gaps and outliers; (2) side-by-side box plots and back-to-back histograms to graphically rule out baseline differences in outcomes between the two study groups; (3) plots of cross-sectional outcome means over time to assess the longitudinal trends within and between the two arms; and (4) scatter plots of change in outcome variables versus change in potential mediator variables. Variables with highly skewed distributions will be transformed. When such transformations are not successful, we will employ generalized linear models in the primary analyses. In addition, we will consider dichotomizing the variables using clinically-relevant cut points.

#### Baseline Analysis

We will test for possible baseline differences in the outcomes and characteristics of the patients and caregivers in the two study groups including but not limited to: patient and caregiver socio-demographic and clinical characteristics, symptom scores, physical and mental functioning, social support, caregiver relationship type (spouse vs. other), and caregiver location (in-home vs. out-of-home). If any significant baseline differences are identified, intervention effects will be estimated using multivariable analysis adjusted for baseline variables as well as stratified analysis using propensity scores.

#### Attrition Analyses

To ensure the validity of findings, baseline characteristics of patients and caregivers who drop out of the study will be compared by study group. We will also summarize and compare reasons for attrition. If any differences are found, the appropriate covariance adjustments or propensity score stratifications will be employed in the primary analysis. To ensure the generalizability of our findings, we will also compare the baseline characteristics of those completing the study against those of subjects who drop out.

#### Primary Analytic Strategy

The primary effect of the intervention will be determined using data obtained at 10 weeks, and the sustained effect will be determined using data obtained at 14 weeks. Cross group comparisons will be made based on an intention-to-treat basis. We will also test the hypotheses with a longitudinal design by fitting a linear mixed-effects modelor a generalized linear mixed effects model to estimate effect averaged over 10 and 14 weeks, adjusting for potential within-person correlation and baseline values of the outcome variables [15].

#### Missing Data

When missing values occur, we will determine whether missingness is random or associated with patient or caregiver characteristics using standard regression models. As long as we do not observe any bias in patterns of dropout or missing data (see attrition analysis above), we will use regression techniques allowing for missing at random. Models will include 10 and 14 week assessment data as longitudinal outcome values, an intervention effect indicator, and baseline values of the outcome variable and other covariates as fixed independent variables. If the analyses indicate that missingness depends on unobserved outcome values(, we will account for missing data using a pattern mixture model [43]. This technique allows the use of data from all participants (not just completers) and provides an unbiased intervention effect estimate.

#### Hypothesis Testing

We will compare summed symptom scores at 10 weeks between the control and intervention groups using a multiple regression model. The model will include a dummy variable for study group assignment as the main independent variable and will be adjusted for baseline symptom scores. While we do not expect the groups to be unbalanced with respect to other potential predictors of the outcome, we will adjust for these if randomization is not fully successful. The parameter estimate of the group assignment dummy variable and its statistical significance will estimate and test for the outcome difference between the two study groups at week 10, adjusting for baseline values of the outcome. A similar approach will be used to test the intervention effect on secondary outcomes (namely physical functioning, service utilization, and chemotherapy adherence). To determine if there is maintenance of the intervention effects on each outcome, we will use a similar regression model using the corresponding measurements at week 14, and a longitudinal model for the outcomes at 10 and 14 weeks.

Our analytic approach for testing Hypothesis 2 will be similar to that described above. We will use multiple regression models to compare group differences in patient-reported social support scores as measured by the Inventory of Socially Supportive Behaviors at 10 weeks, adjusting for baseline values of the ISSB [38]. The model will include a dummy variable for study group assignment as well as the baseline value of the ISSB scores, and the parameter estimate of the dummy variable will estimate the intervention effect on caregivers at 10 weeks. A similar approach will be used to test the intervention effect on caregiver burden and distress.

#### Mediation Analysis

To determine whether the improvements in patient outcomes are mediated by patient self-efficacy and social support, we will employ the approach originally described by Baron and Kenny and more recently further developed by others [44-48]. In addition to the linear models relating outcomes to study group, we will fit two additional models relating mediator to the study group, and the model relating the outcome to the mediator and study group. To establish mediation, the study group effect has to be significant in all but the last model. If in the last model the study group effect is not significant, then we will conclude that complete mediation has occurred. If in the last model the study group effect is reduced, but is still significant, then we will conclude that partial mediation has occurred. To test for mediation, Sobel's test will be carried out, and percent of variation in outcomes mediated by patient or caregiver mediators will be determined [49-51].

## Discussion

Cancer patients undergoing chemotherapy experience many symptoms which they can manage when given the appropriate amount of information. However, self-management may not be sufficient or plausible for everyone. To provide added support to patients for-whom self-management is challenging, we designed Cancer CarePartners-a system offering automated telephonic monitoring of patients with alerts and customized advice to a caregiver via the Web. Our goal is to show that Cancer CarePartners can enhance caregiver support for the patient, so that patients engage in better self-management, and, in turn, experience better symptoms, function, and adherence with chemotherapy, ultimately requiring fewer formal resources during and after treatment. If successful, this intervention will be among the first to demonstrate that caregivers can be formally engaged to improve clinical outcomes.

While we hypothesize that Cancer CarePartners will also reduce caregiver burden and distress by enhancing caregiver knowledge and mastery, it is possible that the opposite may occur; thus, we will closely monitor caregiver outcomes throughout the study. Should caregivers experience increased burden from the intervention, the reasons will need to be explored qualitatively in order to inform the redesign of the system or revise caregiver materials so that they are fully informed of the risks prior to using Cancer CarePartners.

One potential limitation of the study is that not all patients will have access to a caregiver with access to the Web. Additionally, some patients, regardless of their access to a caregiver, may not want to involve others in their care or may prefer a 'human touch' over automation. We will closely monitor reasons for exclusion, refusal and drop out to understand the prevalence of these concerns so as to determine the generalizability of our results. Still, we do not expect that Cancer CarePartners will be appropriate for everyone. We posit that some patients will continue to want or need the support of clinicians by phone or face-to-face. Cancer CarePartners is meant only to help clinicians more efficiently use their time, not replace them.

## Abbreviations

ATSA: Automated Telephonic Symptom Assessment.

## Competing interests

The authors declare that they have no competing interests.

## Authors' contributions

MS, JP, BG, and CWG conceived the study idea. MS, JP, and CWG designed the protocol with contributions from AS for statistical analyses, BG and LN for intervention content, and KC for recruitment protocols and Web site design. MS wrote the first draft of this manuscript, JP and AS revised it significantly, and CWG and LN made minor edits. All authors have given final approval of the version submitted.

## Pre-publication history

The pre-publication history for this paper can be accessed here:

http://www.biomedcentral.com/1472-684X/10/21/prepub
